# Stigma reduction in a disability and mental health programme in Ghana: Lessons in participation

**DOI:** 10.4102/ajod.v14i0.1508

**Published:** 2025-01-31

**Authors:** Maria Zuurmond, Augustina Naami, Lyla Adwan-Kamara, Cathy Stephen, Sapana Basnet, Caroline Vanderick, Mohammed Chantimah, Abigail Nana Asamoah, Daisy Macdonald, Crick Lund

**Affiliations:** 1 Tropical Health, London, United Kingdom; 2 Department of Social Work, University of Ghana, Accra, Ghana; 3 Options Consultancy Services Ltd, London, United Kingdom; 4 Sightsavers, Haywards Heath, United Kingdom; 5 Ghana Education Service, Tamale, Ghana; 6 Centre for Global Mental Health, Department of Health Service and Population Research, Institute of Psychiatry, Psychology and Neuroscience, King’s College London, United Kingdom; 7 Alan J Flisher Centre for Public Mental Health, Department of Psychiatry and Mental Health, University Cape Town, Cape Town, South Africa

**Keywords:** people with disabilities, people with mental health conditions, stigma, discrimination, implementation lessons, participation, inclusion

## Abstract

**Background:**

Stigma is a barrier to inclusion for people with disabilities and mental health conditions. There is increasing recognition of the need to address stigma within disability inclusive programmes, but limited research is available on what are effective participatory approaches to stigma reduction interventions.

**Objectives:**

(1) To document participatory approaches used in the design and delivery of disability stigma reduction interventions in a disability and mental health programme in Ghana and (2) to understand the experience of programme implementers and participants in these processes, with particular attention to the leadership and involvement of people with disabilities.

**Method:**

A mixed-methods study was conducted. The data were drawn from a purposive sample of 20 in-depth interviews (IDIs) with programme staff, partners and key stakeholders, and 12 focus group discussions (FGDs) with self-help group (SHG) members, organisation of people with disabilities (OPD) members, and disability champions. We conducted thematic analysis using deductive and inductive approaches.

**Results:**

The programme adopted several approaches to participation at design and implementation stages, with a focus on the leadership of people with disabilities and people with mental health conditions. The process was seen to promote acceptability of stigma reduction approaches. Providing opportunities for building and strengthening relationships with a wide range of stakeholders was key to successful implementation. Understanding, and engaging with, power dynamics in the local context also provided important benefits.

**Conclusions:**

Participation of people with disabilities and mental health conditions is essential for the design and delivery of stigma reduction programmes in Ghana.

**Contribution:**

This study provided lessons from the field on the value of participation in reducing stigma, and the role of key stakeholders, particularly people with disabilities and mental health conditions.

## Background

Globally, stigma experienced by people with disabilities and people with mental health conditions is pervasive (Mostert 2016; Thornicroft et al. 2022; World Health Organization & World Bank 2011). Stigma can act as a barrier to disability inclusion and the realisation of disability rights (Mostert 2016). This includes being a barrier to accessing education (Banks et al. 2022; United Nations 2019), health services (Kuper & Heydt 2019), and wider participation in society including economic participation (Bonaccio et al. 2020; Thornicroft et al. 2022).

Many factors fuel stigma. In the African context, as in many other regions, a lack of information and understanding on the causes and characteristics of different types of disabilities is identified as a key factor, combined with negative social, cultural and religious beliefs and attitudes (Mostert 2016; Stone-MacDonald & Butera 2012). Disability-related stigma is also pervasive in Ghana, fuelled by deep rooted cultural and traditional beliefs (Awedoba & Denham 2014; Denham et al. 2010; Kassah, Kassah & Agbota 2012; Nyante & Carpenter 2019; Zuurmond et al. 2020a). Evidence shows that stigma, combined with inaccessible environments and institutional barriers, limits participation and inclusion of people with disabilities and people with mental health conditions (Baffoe 2013; Duorinaah et al. 2023; Mfoafo-M’Carthy, Grischow & Stocco 2020; Mfoafo-M’Carthy & Naami 2022).

Despite growing evidence around stigma, and the need to address it, there is still limited evidence of what works to reduce stigma (Heijnders & Van Der Meij 2006; Smythe, Adelson & Polack 2020). We conducted implementation research to explore the use of participatory approaches in the Ghana Somubi Dwumadie (Ghana participation) programme in Ghana, hereafter called ‘the programme’. Implementation research attempts to understand questions related to implementation of a policy or programme. An array of interacting factors can influence implementation, including personnel, resources and contextual factors, which may change over time. Implementation research seeks to capture some of those issues and understand why and how interventions work in ‘real-world’ settings (Peters et al. 2013).

The specific research questions for our study were: (1) What participatory approaches were used in the design and delivery of disability stigma reduction interventions? (2) What was the experience of programme implementers and participants in these processes, with particular attention to the leadership and involvement of people with disabilities?

### The programme

Ghana Somubi Dwumadie was a 4-year disability and mental health programme in Ghana that focused on four areas: (1) strengthening policies and systems; (2) scaling up quality and accessible mental health services; (3) reducing stigma and discrimination and (4) generating evidence to inform policy and practice.

For the stigma reduction element of the programme, a social behaviour change (SBC) strategy was developed to understand and facilitate change in behaviour, societal norms and environmental factors (UNICEF 2023). This focused on three key areas: (1) creating a positive culture of support for people with disabilities, (2) increasing the use of positive disability and mental health language and (3) ensuring duty bearers enforce relevant policies and laws. Key audiences targeted were the media, community members, family members, religious, traditional and community leaders, and people with disabilities, including people with mental health conditions. The strategy was primarily implemented through grants to organisations of persons with disabilities (OPDs), and Women’s Rights Organisations and Civil Society Organisations. One of the approaches for implementing activities was the use of ‘Disability Champions’, that is, people selected to advocate for, and engage in, the design and delivery of activities, and paid a small stipend. While there was some variation in the term used (e.g. ‘Disability Ambassadors’), for the purpose of this article, we use ‘Disability Champions’ throughout.

While it was stated that ‘participatory approaches’ would be applied in the programme, this has not yet been rigorously evaluated. The purpose of this study is therefore to better understand what participatory approaches were used during the implementation of the programme.

## Research methods and design

The study used a design suited to implementation research, as defined earlier. This involved a mixed-methods approach, combining data from in-depth interviews (IDIs), focus group discussions (FGDs) and a review of programme documentation. In terms of exploring the experience of people with disabilities, we applied an intersectional lens, where intersectionality is defined as acknowledging the multiple social categorisations of a person’s identity, how these interact and can result in different experiences of advantage or disadvantage (Moodley & Graham 2015).

### Data collection

One-day training on reviewing and use of the data collection tools was conducted in Accra. Data collection was undertaken by two research teams, each consisting of a lead senior researcher and research assistant, and inclusive of two people with disabilities, who were familiar with the national and local disability context. Field work was conducted between 17th June 2023 and 27th June 2023.

The IDIs were conducted using semi-structured interview guides that were adapted for each group, including government institutions, local non-governmental organizations (NGOs) and OPDs, traditional and religious leaders, staff and grantees. The FGDs were undertaken with 3–10 participants with a range of disability self-help group (SHG) members, disability champions and members of OPDs. The topic guides included questions about the programme design process; what types of activities individuals and/or organisations were involved in; the nature of that participation; what they felt worked well and why; challenges and areas to improve. Similar questions were tailored to the delivery of the interventions. Questions were included on how men and women with disabilities were engaged in both the design and delivery processes, which included an understanding of selection processes and facilitators and challenges in fulfilling those roles. All interviews were conducted in participants’ preferred language; local language and sign language interpreters were employed to support the study, as needed. Interviews took between 60 min and 90 min. Detailed notes for all IDIs and FGDs, and additionally, recordings were made and used as necessary to check or clarify issues, if required. All data were stored on password-protected computers. Data were anonymised before analysis and when reporting the results of this study (Sightsavers 2023).

### Study setting and sample

A purposive sample was used, with the following criteria: a geographical spread, a focus on areas with ongoing work, the inclusion of people with different types of disabilities, including people with mental health conditions, a gender balance, and consideration of logistical and safety constraints.

For the purpose of this article, we drew on a subset of the data concentrating on two partner organisations where stigma reduction activities constituted the main focus of their work. For the purpose of anonymity, we describe these as ‘Organisation A’ and ‘Organisation B’.

### Analysis

A thematic analysis of the qualitative data was conducted (Pope, Ziebland & Mays 2000). An initial coding frame was developed linked to the topic guide outline and a priori themes that emerged from the review of secondary programme documentation. We used a combination of deductive and inductive approaches to the analysis, with new themes and sub-themes being added and modified in an iterative process as the two research teams discussed emerging findings during fieldwork and analysis. Preliminary findings were shared during a participatory stakeholder workshop at the end of field work, and further nuances were made to key themes. The lead researchers independently conducted their own data analysis using the framework and were in contact regularly to compare and contrast findings across sites. NVivo software and Word were used to manage the final data analysis.

### Ethical considerations

Ethical clearance was received from the University of Ghana’s Ethics Committee for Humanities (ECH 162/22-23). Participation in the study was voluntary and interviewees could opt out at any time and were informed that they were free to stop the interview at any time. Informed consent to participate in and to record interviews was obtained from all participants.

## Results

In total, the sample consisted of 20 IDIs and 10 FGDs, which took place across five regions in Ghana. For the details of the sample characteristics, see Table 1.

**TABLE 1:** Sample characteristics.

Type of organisation/group	Region	M	F	Disability/mental health condition and/or organisational focus
Programme staff	National	5	3	-
OPDs – partner/grantee	National, Northern, North-East, Volta	10	9	Physical disability (4), visual impairment (3), hearing impairment (4), albinism (2), mental health focus
Mental Health NGO Network (inclusive of OPDs)	Volta	5	3	Mental health focus
SBC grantee (Organisations A and B)	National North East, Savanah, Volta	7	9	Physical disability (2)
Faith-based organisations	Volta/North East	4	1	-
Traditional authority/leaders	Volta/North East	3	2	-
Government institution (e.g. health, social welfare)	Volta	6	1	Mental health focus
Self-help group members	Volta/North East	31	12	Physical disability (23), visual impairment (6), epilepsy (1), HIV (4), multiple disability (1), caregiver (14)
Disability champions	Volta/North-East, Savanah	13	7	Physical disability (6), albinism (1), visual impairment (2)

NGO, non-governmental organization; OPD, organisations of persons with disability; SBC, social behaviour change; HIV, human immunodeficiency virus; M, male; F, female.

This results section is divided into two connected parts. The first part describes where and how participatory processes were used in the design and delivery phases. The second part explores the experience of programme implementers and participants in these processes. The key themes are overlapping and include: (1) the empowerment of persons with disabilities, with subthemes of knowledge, confidence building, self-esteem and internalised stigma, (2) intersectionality, (3) wider participation of key stakeholders and subthemes of relationship building and engaging with people with power and (4) acceptability.

### Participation steps in programme design and delivery

People with disabilities, including people with mental health conditions, played a variety of roles in the design stages of the programme’s stigma reduction work. Other key stakeholders, relevant to the context, were also invited. Step one was a formative research study with a wide range of stakeholders, which included men and women with disabilities and/or mental health conditions. This explored the lived experiences of stigma, as well as the major drivers. The results from the formative research fed into two co-creation workshops, which were conducted, firstly to develop an SBC strategy for stigma reduction, and secondly, to explore how to operationalise the strategy so that it was relevant to the local context. People with disabilities and people with mental health conditions, mainly in leadership roles, were key participants in the workshops, selected from SHGs and OPDs.

**FIGURE 1:**
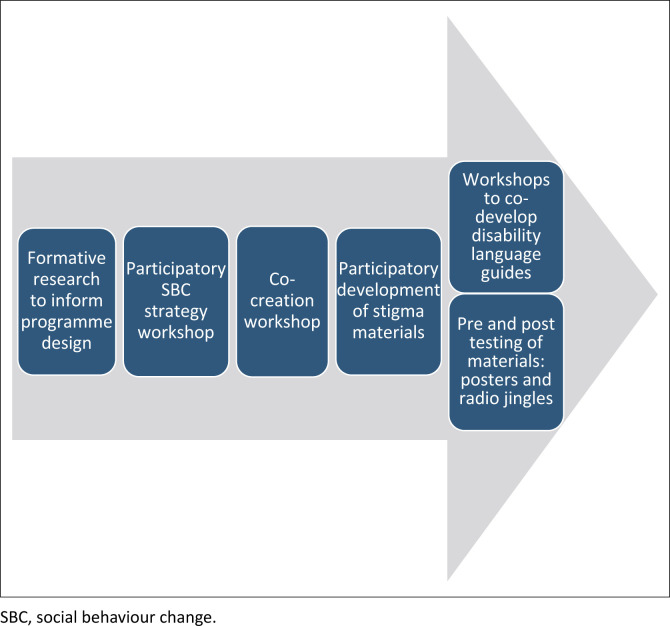
An illustration of key participation points in the design phase.

The participatory approach continued as the programme evolved. For example, regional workshops were conducted for designing a Disability Language Guide. Stigmatising language had been identified as a key driver of stigma, and the involvement of people with disabilities in the co-development of the guide was identified as an important process. People with disabilities were also engaged in a three-stage process of pretesting, adapting and reviewing posters and radio jingles in local communities. See Figure 1 for a summary of key participation steps in the design and delivery.

In terms of delivery, OPDs were one conduit for delivering the programme, alongside women’s rights organisation, and civil society organisations. A common approach was for them to employ disability champions, who were selected community members who had a variety of roles in stigma reduction; an educational role, an advocacy and influencing role, and responsibility for reporting on issues of abuse, described as an ‘enforcement’ role.

### Empowerment of people with disabilities

#### Building knowledge, confidence and self-belief

As a result of their participation, people with disabilities in disability champion roles and/or within OPD positions, who were interviewed, were positive about their participation in stigma reduction activities, most commonly reflecting on increased knowledge and confidence to be engaged in activities. This is illustrated here by a female disability champion from the Savannah Region who had participated in the co-design of a ‘Positive Disability Language’ guide; she then later felt she could adapt and use the material in her local setting, ‘I was so excited; I was so happy …. I then walked into the radio station to talk about this work’.

In the Volta Region, OPD members reflected on increased awareness and feelings of dignity as a result of their engagement in the language guide design:

‘We ourselves coming together to deliberate on derogatory names and developing a Positive Language Guide has given us some awareness about how these names were really affecting us negatively, affecting our dignity and the need to advocate for our rights.’ (FGD, Partner Organisation, Volta Region)

Several different Positive Language Guides were developed, by grantees in different regions, which allowed them to be tailored to the local language and encouraged local engagement on the tool. They were also a popular tool to use for the interactive programme on the radio. For example, one of the grantees had a radio programme called Dignity Hour with people with disabilities as resource persons speaking on the radio.

This growth in confidence, and self-belief, was also reflected as a consistently strong theme in the secondary programme data, illustrated here by a final change story:

‘At first, I was feeling shy to participate in community and family gatherings. I thought they will not listen to my views based on several experiences. Through this project, I’m empowered to be involved in activities in my family and community.’ (woman with a disability, secondary data, small grants evaluation)

#### Internalised stigma

Interlinked with the issue of empowerment and building self-confidence was the challenge of internalised stigma, or more commonly described by staff as ‘self-stigma’. This was mainly identified in staff and partner interviews as one of the barriers they identified when engaging with leadership of people with disabilities and mental health conditions, and something that they felt needed to be better understood and addressed in order to maximise their participation. This was described here by one grantee:

‘Self-stigma was high throughout the implementation of the project; we had to constantly talk to the disability champions to give them confidence in themselves. [*What do you mean by self-stigma?*]. That you yourself are looking at your condition – I have self -pity, I don’t belong, I make myself less of a person with my thoughts – whereas if you respect yourself then you have a voice.’ (IDI, Grantee, NE Region)

The review of secondary programme documentation similarly highlighted internalised stigma as a challenge faced in encouraging participation. For example, a survey of 159 people with disabilities as one component of a programme evaluation illustrated that ‘being worried that others will find out they had a mental health or disability’ (66%) or ‘feeling ashamed of their condition’ (49%) were the main forms of stigma that existed at the end of the projects.

### Intersectionality and participation

Overall, there were lower levels of participation of women with disabilities, and while there was some improvement over time, such as setting targets for women’s participation in trainings, and the engagement with female-focused OPDs, it still remained an issue. Identified challenges included limited available time, given other domestic and caring roles within the family, and lower overall representation of women in leadership positions within OPDs and SHGs.

People with hearing impairments were also less likely to be well represented, with the exception of targeted engagement with the national deaf OPD. The commonly held view across all sites was that what limited their participation was not stigma but the dearth of sign language interpreters. This deficit of sign language interpreters was identified as a national problem, and one key stakeholder argued this was an example of ‘major discrimination’, preventing the meaningful engagement of people with hearing impairment. For people with a mental health condition, at a structural level, there was some apparent progress towards better linkages with the regional OPD umbrella organisations, but otherwise their representation was still limited, for example, in roles such as disability champions.

Other characteristics such as poverty, type of disability, and position and relationships in society were also reported to shape the experience of people with disabilities in their level of participation and ability to implement. This is illustrated here by two different experiences of disability champions; one man with a physical disability, a teacher, had been secretary of the regional disability network for 14 years, a member of the District Assembly Common Fund Committee, and owned his own motorbike. He was very active in his role, leveraging his wide range of contacts to support his work, including informal meetings with key people to support the stigma reduction work. In contrast, at a second very rural site, a young woman with albinism explained how she was a single mum, struggling financially with no job aside from the small stipend for her disability champion role, also supporting her sister’s family as well as her own two children, and had no access to her own transport. She had limited networks to draw upon. While she was committed to her role, she expressed her limited capacity at times to implement activities and requested more training and support.

### Broad participation of key stakeholders

While there was a focus in the programme on the participation of people with disabilities and mental health conditions, a key part of the strategy was also engagement with and building alliances with a broader range of stakeholders, including religious leaders, caregivers, NGOs, traditional authorities and government implementing agencies. Key overlapping subthemes here were the benefits of building strong relationships and engagement with people in power.

Opportunities for building and strengthening relationships were afforded in various planning and co-design meetings, for example, the use of ‘interface’ meetings, which were meetings that were facilitated between SHGs and key officials in local government assemblies. These spaces were seen to provide an important platform to have contact with, understand and engage with individuals and/or organisations who typically had power at various levels in the community, namely traditional leaders, religious leaders, the media and various local government organisations and platforms:

‘What we gave the [*disability*] champions was a structure to engage with the key players.’ (IDI, Organisation A, grantee staff, NE Region)

The benefits of building these relationships were highlighted particularly by people with disabilities, for example in their role as disability champions; ‘through this togetherness more senior people attended meetings’, people were more likely to listen to them ‘if they were on your side’:

‘I will go around on my bicycle from place to place – markets, church, youth groups. I will meet traditional leaders as they are powerful.’ (FGD, Disability Champions, Savannah Region)

The secondary programme data on the co-development of the disability language guides also illustrated how having co-design workshops enabled people with disabilities an opportunity to directly challenge experts, including academics and local educators and explain what local language words and descriptions around disability and mental health were acceptable to them, resulting in changes to the material.

This building of relationships with the relevant government or traditional authorities was also identified as key to their adoption of the stigma interventions. This was most notably observed in the Volta Region where key stakeholders played a particularly strong role in implementation of the stigma work, which included the use and reinforcement of sanction mechanisms, such as the application of fines, and upholding of the law, one of the key strands of stigma reduction work:

‘And I told the people with disabilities are also human like you. They eat, drink and do other things like you, so if anyone call them names like ‘Pozo’ [*derogatory term for cripple and nickname given to a Ghanian musician with a disability*] the person would be charged GHC500.00 [*45 dollars*].’ (IDI, traditional leader, Volta Region)

‘Previously I saw Commission on Human Rights and Administrative Justice [*CHRAJ*] as an organisation not to get closer to, but it [*the project*] has brought us to those who are powerful.’ (FGD, Disability Champions, Savanah Region)

The different approaches to selecting disability champions illustrated some lessons in terms of the benefits of fostering different types of relationships within the programme. In Organisation A, all disability champions were people with disabilities and people with mental health conditions, selected in consultation with the regional chapter of the national disability umbrella organisation, and state organisations. They were largely chosen from those in leadership roles in OPDs and SHGs. One of the perceived strengths of this approach was continued close engagement with their membership organisations, where members, for example, described them as being a ‘mouthpiece’ for them. In one site, however, a weaker relationship with the regional disability network resulted in less overall support.

In contrast, in Organisation B, the disability champions were a mix of both people with disabilities and mental health conditions (40%) and other community members (60%). Organisation B leveraged strong relationships with traditional leaders, largely in the rural areas, to recommend community members as disability champions. The identified advantages were that local leaders were generally found to be willing to give ongoing support, which also enhanced community acceptance of the work. Community members elected to the role were teachers, District Assembly and Committee members, and religious and opinion leaders. They were generally influential people in the community who leveraged their connections and power in different settings: town hall meetings, schools, radio stations and community information centres. A summary of these different models of disability champions is provided in Table 2.

**TABLE 2:** A summary of models of disability champions.

Organisation	Total DCs	People with disabilities	Gender	Selection process	Key advantages
A	38	All	9 F/29 M	Consultation with umbrella OPD and state institutions	Close engagement with local OPD and potential support from regional umbrella network
B	44	17 plus teachers, assembly members, religious leaders	19 F/25 M	Consultation with local traditional leaders	Endorsement and support of activities by local leaders, and leverage of their networks

F, female; M, male; OPD, organisations of persons with disability; DC, disability champion.

Overall, Organisation B was particularly strong in their engagement with partners. These strong relationships were attributed to several factors; having a long history, almost 20 years, of the organisation working at the grassroots in the region on disability rights, existing strong networks and trusted relationships to build upon, and involving partners from the start of the design process. All of this contributed to greater active participation of other stakeholders in adopting and implementing the stigma work, illustrated here by a quote from a government department:

‘We as a department has worked with them for a very long time. And we know they play a major role in the disability sector, supporting and complementing what the government does. And with the introduction of the social behaviour change [*strategy*], when they started, they also called us to inform us about what they were doing and if we had any input to make. … I used it [*the Positive Language Guide*] to sensitise our officers and they are also using it.’ (IDI, Government institution, Volta Region)

### Acceptability

Another recurring theme was that stigma reduction materials and approaches are rendered more acceptable by the participation of people with disabilities and mental health conditions and other stakeholders. Greater acceptability was reflected in the high levels of satisfaction expressed about the suitability and culturally appropriate stigma materials and approaches for the local audience. The role of people with disabilities directly engaging with other key stakeholders meant that traditional views could also sometimes be challenged, and more inclusive and acceptable language and images then adopted. For example, the development of the language guides in a co-design workshop afforded a platform for people with disabilities to directly challenge experts, such as academics and other educators, and explain directly what types of terminology were more acceptable to them.

‘The posters made an impact on the community as the community sees their members with disabilities telling them in their own language to stop name calling which sends a signal. So, more materials should be translated into the local language.’ (KII, partner organisation, Region B)

Another participatory approach adopted was the pretesting of materials for the production of posters, radio jingles and language guides. This was strongly implemented at one site (Organisation B) where a combination of people with disabilities and mental health conditions, and a range of local partners, including local traditional leaders, were engaged in a three-step review process. The poster images in this region were then changed from the original ones, to preferred local images, combined with local languages used on radio jingles.

A consistent sub-theme was that the participation processes fostered greater ownership, thus strengthening the wider adoption and use of the materials. This was illustrated by the example of a female traditional leader who was engaged in the design process and then placed a poster on her palace wall, and used it to prompt discussion around stigma and disability. Other examples included local chiefs contributing to the language guides and promoting the stigma resources on the radio and via local community information centres. Importantly, there was also evidence of adoption of the materials by Christian and Islamic religious leaders in this same region.

However, in a second site, limited time was highlighted by Organisation A as one of the tensions in adopting quite lengthy participatory processes, which resulted in more limited stakeholder engagement. As a result, there were fewer adaptations of the materials and less take up and application of the stigma materials.

## Discussion

This implementation research has illustrated the processes of implementing a participatory stigma reduction intervention for people with disabilities and mental health conditions in Ghana and explored their experiences of this engagement. The programme demonstrated how it is possible to provide meaningful opportunities for people with disabilities and mental health conditions, to engage in both design and implementation of a stigma reduction strategy. This aligns with the core general principle of the United Nations Convention on the Rights of Persons with Disability (UNCRPD), to uphold ‘full and effective participation and inclusion in society’ and the importance of being ‘actively involved in decision-making processes about policies and programmes that affect their lives’ (United Nations 2006).

While there is limited evidence of what works in stigma reduction, research has shown the importance of maximising social contact with people with disabilities and mental health conditions (Adu et al. 2022; Heijnders & Van Der Meij 2006; Smythe et al. 2020). In this article, it was evident that through promoting participation of people with disabilities and mental health conditions, they were provided with opportunities for direct engagement with community stakeholders and thus arguably have greater visibility. This is likely to help contribute towards stigma reduction as part of the process of programme implementation.

One of the identified benefits of a participatory approach shown in our study was enhanced acceptability of the stigma-related materials, where acceptability is defined as satisfaction, intent to use, perceived appropriateness and fit within organisational culture (Bowen et al. 2009). Other studies have similarly illustrated how direct and expert knowledge of their issues can help identify and address their unique needs (Mfoafo-M’Carthy & Naami 2022) and support an approach that is more culturally relevant (Hartley et al. 2009).

This study also demonstrated the identified benefits of participation in terms of improved knowledge, confidence and self-esteem of people with disabilities. This can be described as personal empowerment, or ‘power within’, adopting the model of Rowlands (1997) in her exploration of the different dimensions of empowerment. Heijnders and Van der Meij (2006) in their review of disability-related stigma reduction interventions also detail that an approach which targets the ‘intrapersonal’ level is vital for individual empowerment, who is then in a better position to effect change.

Interlinked with this notion of facilitating personal empowerment, we identify the issue of internalised stigma as a challenge to participation. Other studies have shown how internalised feelings of oppression and shame can result from social stigma, and commonly experienced among people with disabilities (Rohwerder 2018), and people with mental health conditions (Ali et al. 2012; Brohan et al. 2010). This highlights the need to further strengthen this stigma component in order to maximise participation and in turn empowerment.

The study also illustrated that engagement with power holders provided a range of additional benefits in the implementation of the interventions. We know from other studies on civil society participation the importance of understanding and harnessing those who have power over economic, social or political factors in order to effect change (Gaventa 2005). In terms of influencing social stigma, the participation of opinion leaders is likely to be important in changing social norms (Rohwerder 2022; Van Brakel et al. 2019). It has also been argued that to reduce stigma there needs to be a better understanding of the relationship between stigma and power, in order to change the balance of power between those who stigmatise and those who are stigmatised (Link & Phelan 2014). In the context of Ghana, a study with caregivers, often single mothers, also illustrated their limited power over social and political processes and the value of harnessing other gatekeepers to effect change (Zuurmond et al. 2020b).

Finally, in our study we illustrate that different characteristics such as gender, type of disability and poverty intersect to shape peoples’ experience and level of participation. The lower overall engagement of women with disabilities reflects the global literature (Moodley & Graham 2015; United Nations Women 2018) and the fact that men still dominate leadership positions of the SHGs and the OPDs is also consistent with the literature in the Ghanaian context (Naami 2014). Deaf people were also less likely to participate, held back by the dearth of sign language interpreters. The lack of availability, and sometimes limited proficiency of sign language interpreters, has been demonstrated in other studies in Ghana (Mprah et al. 2024) and this is further complicated when deaf people themselves are not proficient in sign language (Duorinaah et al. 2023). This highlights the importance of applying an intersectional lens in programme design, with targeted approaches to increase the capacity and participation of women with disabilities, including women-led OPDs, and avenues to further strengthen the participation of people with mental health conditions and people who are deaf.

### Strengths and limitations

A strength of this implementation research was the inclusion of researchers with knowledge of the local context, as well as lived experience of disability in Ghana. Both research teams constantly compared and contrasted findings across field sites to maximise the opportunity for reflexivity and minimise bias (Darawsheh & Stanley 2014). Nevertheless, some of the authors were also involved in the design and delivery of the programme, and this may have influenced their perspectives and interpretations of the experiences captured in this study. The methodology used a purposive sample and the findings are therefore not generalisable but the triangulation of findings between FGDs, IDIs and secondary data, and a final workshop to share emerging findings with key stakeholders, strengthened the validity of the data. There was limited representation of people with mental health conditions and people with hearing impairments, even though for the latter group, sign language interpreters were available. This may have biased findings and would need to be addressed in future research. In future it would also be useful to gather more in-depth data on disability champions in order to better understand the dynamics and intersectional factors that shape their work on stigma reduction.

## Conclusion

Stigma is a barrier to inclusion for people with disabilities and people with mental health conditions. The adoption of participatory approaches that promote the engagement of people with disabilities and people with mental health conditions can support elements of their understanding of how power dynamics work in the local context and is key; building relationships with and encouraging the participation of people with disabilities and mental health conditions can offer many benefits for implementing stigma reduction.
